# Targeting Key Players of Neuroendocrine Differentiation in Prostate Cancer

**DOI:** 10.3390/ijms241813673

**Published:** 2023-09-05

**Authors:** Irene Zamora, Michael R. Freeman, Ignacio J. Encío, Mirja Rotinen

**Affiliations:** 1 Department of Health Science, Public University of Navarre, 31008 Pamplona, Spain; 2 Departments of Urology and Biomedical Sciences, Cedars-Sinai Medical Center, Los Angeles, CA 90048, USA; 3 Department of Medicine, University of California Los Angeles, Los Angeles, CA 90095, USA; 4 Instituto de Investigación Sanitaria de Navarra (IdiSNA), Navarre Institute for Health Research, 31008 Pamplona, Spain

**Keywords:** prostate cancer, lineage plasticity, neuroendocrine transdifferentiation, targeted therapy

## Abstract

Neuroendocrine prostate cancer (NEPC) is a highly aggressive subtype of prostate cancer (PC) that commonly emerges through a transdifferentiation process from prostate adenocarcinoma and evades conventional therapies. Extensive molecular research has revealed factors that drive lineage plasticity, uncovering novel therapeutic targets to be explored. A diverse array of targeting agents is currently under evaluation in pre-clinical and clinical studies with promising results in suppressing or reversing the neuroendocrine phenotype and inhibiting tumor growth and metastasis. This new knowledge has the potential to contribute to the development of novel therapeutic approaches that may enhance the clinical management and prognosis of this lethal disease. In the present review, we discuss molecular players involved in the neuroendocrine phenotype, and we explore therapeutic strategies that are currently under investigation for NEPC.

## 1. Introduction

Prostate cancer (PC) is a public health concern with a global impact, affecting millions of men worldwide [1]. Among men in the United States, PC is the most diagnosed cancer, other than skin cancer, and ranks second as the leading cause of cancer-related mortality [2].

Approximately 90–95% of diagnosed PC are adenocarcinomas with a luminal phenotype that arise as an androgen-driven disease [3,4]. Therefore, androgen deprivation therapy (ADT) is the standard first-line treatment approach for PC. Despite initial responses to ADT, the acquisition of resistance mechanisms is nearly universal, leading to metastatic castration-resistant prostate cancer (mCRPC), a lethal form of the disease [5,6]. As a result, novel AR signaling inhibitors (ARSIs), such as the AR antagonist enzalutamide or the androgen biosynthesis inhibitor abiraterone, have been introduced to clinical practice [7]. These next-generation drugs have improved the overall survival of mCRPC patients [8,9], but unfortunately, resistance ultimately arises. The majority of mCRPC tumors exhibit a reactivation of androgen receptor (AR) signaling through a variety of mechanisms, including AR amplification, activating mutations [10,11], AR splice variants [12] or ligand-independent activation [13,14]. Other tumors overexpress the glucocorticoid receptor (GR) pathway, which circumvents AR blockade [15]. In addition, under prolonged AR pathway inhibition, tumors can also progress to an AR-indifferent state, which occurs in 15–20% of mCRPC tumors [16]. One mechanism behind this process is the histologic transformation from adenocarcinoma to a poorly differentiated neuroendocrine (NE) carcinoma with absent or low AR expression levels [16]. This lethal subtype of PC is known as neuroendocrine prostate cancer (NEPC).

The management of NEPC is challenging as there is no standard effective therapy. NEPC cells are insensitive to therapies targeting the AR pathway [17], and patients are treated with systemic chemotherapy [18]. Nevertheless, the prognosis of NEPC patients remains poor, with a median survival of only 10 months [19]. Because of these important clinical implications, metastatic biopsies in any patient with mCRPC may be considered if there is clinical suspicion of induced NEPC transformation [20,21]. The NE phenotype is characterized by unique morphological features of circulating tumor cells (CTCs) [22] and genomic/epigenomic alterations in cell-free DNA [23]. These characteristics can be detected through liquid biopsies, providing an opportunity for early and non-invasive identification of NEPC patients during adenocarcinoma progression. In this way, advanced techniques, such as next-generation sequencing (NGS), have demonstrated clinical utility in NEPC diagnosis and monitoring [24].

Recent progress in understanding the biology of NEPC has led to the development of experimental approaches that address its unique molecular characteristics, holding the potential to improve the treatment and clinical management of this aggressive disease. In this article, we review the collection of molecular events contributing to NEPC onset and progression and current treatment strategies to target them.

## 2. Molecular Mechanisms Underlying NEPC and Key Factors in Neuroendocrine Differentiation

De novo NEPC accounts for less than 1% of all PC at the time of diagnosis [25]. NEPC more commonly emerges as a treatment-induced tumor, where PC cells undergo a phenotypic switch to alternative lineage programs as an adaptive mechanism to evade therapies. Besides ADT or ARSIs [4], NEPC can also emerge under radiotherapy [26] or chemotherapeutic regimens [27].

**Figure 1:**
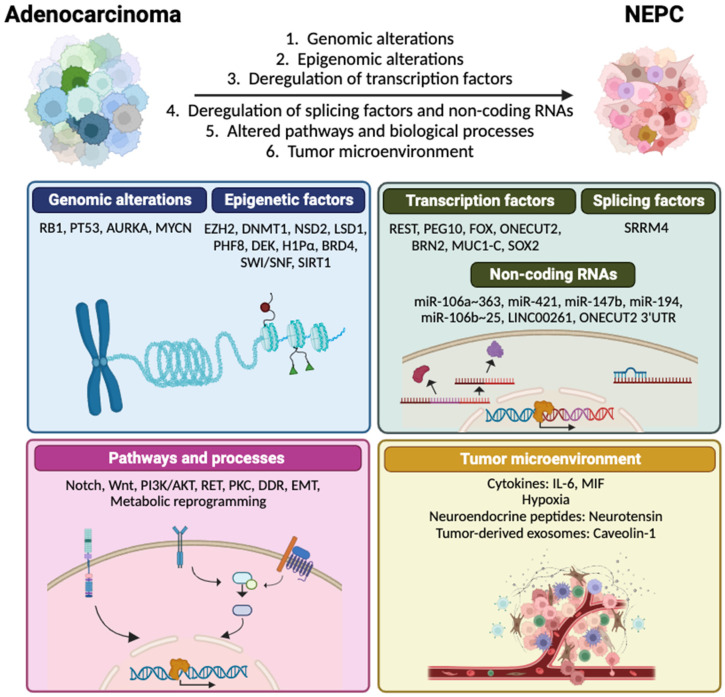
Molecular events and key factors contributing to NEPC transdifferentiation from prostate adenocarcinoma. DDR: DNA damage response, EMT: epithelial-to-mesenchymal transition, MIF: macrophage migration inhibitory factor.

The precise mechanisms promoting NEPC remain largely unknown, and it is still unclear whether it develops through direct transdifferentiation or an intermediate stem-like cell state [4]. However, a wide range of genomic and epigenomic alterations, transcriptional variations, disruptions in other molecular pathways and alterations within tumor microenvironment (TME) are considered drivers that fulfill temporal roles that contribute to NE transdifferentiation [28] (Figure 1).

### 2.1. Genomic Alterations

Several genomic alterations are enriched in NEPC. One prevalent event is the loss of RB1 (Retinoblastoma 1) and/or TP53 [29]. These tumor suppressor genes are key facilitators of lineage plasticity [30,31]. While the aberrations in RB1 and/or TP53 are not directly responsible for the induction of NE genes, their loss results in the upregulation of other factors essential for NE differentiation, such as EZH2 (Enhancer of Zeste Homolog 2) and SOX2 (SRY-box transcription factor 2) [31].

Amplification of MYCN and AURKA (Aurora Kinase A) genes has also been observed in NEPC, with amplification of both genes seen in approximately 40% of NEPC cases and 5% of adenocarcinoma tumors [32]. MYCN and AURKA cooperate in a positive feedback loop to induce an NE phenotype in PC cells [32,33]. The precise underlying mechanism by which AURKA drives NEPC remains unclear, but its amplification is associated with deregulated proliferation and aggressive tumor behavior [34]. MYCN directly binds to the promoters and drives the expression of NE genes such as NSE (Enolase 2) and SYP (Synaptophysin) and suppresses the AR and its transcriptional program [32].

### 2.2. Epigenomic Alterations

The distinct cell lineage phenotype observed in NEPC compared to adenocarcinoma cannot be fully attributed to genomic alterations. This finding implies the existence of additional mechanisms, including epigenetic changes, as significant contributors to NEPC development and progression [35,36]. Epigenetic regulators can modulate the chromatin structure, DNA methylation patterns and histone modifications, thereby establishing a chromatin landscape that promotes increased cellular plasticity and NE differentiation [37].

EZH2 is frequently overexpressed in NEPC and has been confirmed as a master regulator of NE differentiation of PC cells [29,32,38]. As a component of the PRC2 (Polycomb Repressive Complex 2) complex, EZH2 carries out the methylation of histone H3 at lysine 27 (H3K27). In addition to its interaction with MYCN to suppress AR signaling [39], it also regulates other factors with important roles in NEPC progression, such as DNMT1 (DNA Methyltransferase 1) [40] and NSD2 (Nuclear Receptor Binding SET Domain Protein 2) [41,42].

Certain histone lysine demethylases contribute to NEPC transformation. LSD1 (Lysine-specific demethylase 1), also known as KDM1A, possesses dual activity in demethylating histone H3 at lysine 4 (H3K4) and lysine 9 (H3K9). LSD1 has been identified as an important regulator of AR transcriptional activity [43] and promotes NEPC cell survival by repressing TP53 signaling [44]. In NEPC, a neuronal-specific isoform of LSD1 called LSD1 + 8a is specifically overexpressed and is considered a key player in promoting NEPC differentiation [45]. The histone demethylase KDM7B, known as PHF8 (Plant Homeodomain Finger Protein 8), is also a driver of NEPC [46,47]. PHF8 upregulates FOXA2 (Forkhead box A2) by demethylating and removing repressive histone marks on FOXA2 promoter, which subsequently regulates the expression of genes involved in NE lineage plasticity [47].

The DNA topology modulator DEK and the chromatin crosslinking protein HP1α (Heterochromatin protein 1α) are other key epigenetic regulators in NEPC pathogenesis. In prostate adenocarcinoma models, HP1α exhibits an early upregulation after castration, and its expression increases gradually, reaching its highest level in fully developed NEPC [48]. HP1α silences the AR and REST (RE1 Silencing Transcription Factor), two transcription factors found downregulated in NEPC [48]. Following the elevated levels of HP1α, there is a subsequent increase in DEK expression, which remains consistently high during the transition to NEPC. Importantly, this post-castration increase in DEK expression is not observed in other adenocarcinoma models that give rise to AR-positive relapsed cancers [49].

The bromodomain and extra-terminal (BET) family of proteins are important chromatin readers involved in NEPC. Among the BET proteins, BRD2/3/4 have been identified to directly interact with the AR [50]. The isoform BRD4 cooperates with E2F1 (E2F Transcription Factor 1), usually activated after RB1 loss [51], to initiate the AR-repressed NEPC lineage plasticity program [52]. Additional evidence links BRD4 to the epithelial-to-mesenchymal transition (EMT), a process closely associated with NE differentiation [53].

Other epigenetic components that participate in lineage plasticity are members of the SWI/SNF (SWItch/Sucrose Non-Fermentable) complexes. Several subunits of the SWI/SNF complexes show alterations in the setting of CRPC-NE, with a notable upregulation of SMARCA4 associated with a more aggressive clinical course [54]. Additionally, some histone deacetylases (HDACs), such as Sirtulin 1 (SIRT1), have been implicated in NE transdifferentiation. SIRT1 is induced by ADT and promotes NEPC through activation of AKT signaling [55].

### 2.3. Deregulation of Transcription Factors

Alteration in the expression or activity of a number of transcription factors that enhance or repress the NE lineage phenotype has been documented. Suppression of REST expression is a hallmark feature of NEPC [56]. REST functions as a transcriptional repressor of neuronal genes [57] and, thus, in NEPC, loss of REST expression leads to the upregulation of NE genes [56]. REST is also involved in EMT and the acquisition of cancer stem cell (CSC) characteristics promoting NE reprogramming of PC cells [58].

The placental gene PEG10 (Paternally Expressed 10) is directly repressed by the AR in prostatic adenocarcinoma, and consequently, its expression is significantly elevated in NEPC [59]. Using the LTL331 transdifferentiation model that includes the transition from adeno- to NEPC, PEG10 was identified to be a driver of the NE phenotype [60] that follows the HP1α expression pattern previously mentioned, with an early upregulation after castration and a subsequent increase in intensity in terminal NEPC [28,60].

The FOX transcription factors FOXA1 (Forkhead box A1), FOXA2, FOXB2 (Forkhead box B2) and FOXC2 (Forkhead box C2) are fundamental in NEPC development. FOXA1 has been described as an inhibitor of PC NE differentiation that is lost with the progression of mCRPC [61]. Conversely, FOXA2, FOXB2 and FOXC2 promote NEPC through activation of the KIT pathway, Wnt signaling and EMT/CSC, respectively [62,63,64]. The adeno-to-NE lineage transition mediated by FOXA2 requires cooperation with HIF-1α (Hypoxia Inducible Factor 1-alpha), a process facilitated by the ubiquitin ligase SIAH2 (Siah E3 Ubiquitin Protein Ligase 2) [65].

The developmental transcription factor ONECUT2 (One Cut Homeobox 2) also synergizes with HIF-1α [66] to drive epithelial to NE differentiation. ONECUT2 expression is significantly higher in clinical NEPC samples compared to benign prostate, primary adenocarcinoma and mCRPC-adenocarcinoma. It has been demonstrated that the repression of ONECUT2 by REST is released in NEPC, resulting in the activation of ONECUT2, which thereby regulates the expression of other key players of NE differentiation, including PEG10 upregulation and FOXA1 inhibition [67].

BRN2 (Brain-specific homeobox/POU domain protein 2) is a master regulator of neuronal differentiation, which is highly expressed in NEPC compared with adenocarcinoma tumors. BRN2 overexpression is sufficient to induce NE markers and aggressive growth of PC cells [68]. BRN2 is directly suppressed by AR signaling [68] while its expression is induced by MUC-1C (Mucin-1), which also regulates other factors, such as SOX2 and the NF-κB (Nuclear Factor Kappa B) pathway, to promote epigenetic reprogramming and EMT contributing to NEPC [69].

SOX2 and LIN28B (Lin-28 Homolog B) pluripotency factors are highly expressed in NEPC [68,70] and confer a CSC phenotype to PC cells, facilitating the acquisition of phenotypical changes such as NE differentiation [70,71,72]. SOX2 expression has been shown to be repressed by the AR and induced by BRN2, MUC-1C and E2F factors [68,69,73]. SOX2 seems to have a repressor role, as it causes a decrease in the expression of adenocarcinoma-specific genes in NEPC via LSD1-mediated global epigenetic modulation [74].

### 2.4. Deregulation of Splicing Factors and Non-Coding RNAs

The splicing factor SRRM4 (Serine/Arginine Repetitive Matrix 4) is upregulated in NEPC [32,57]. SRRM4 can impart NE features to adenocarcinoma cells, an effect that is exacerbated by the loss of RB1 and TP53 and/or the use of ARSIs [75]. Among the key factors that SRRM4 regulates is REST, resulting in the generation of a truncated and inactive form (REST4) that lacks the transcriptional repressor domain [57,75], and LSD1, promoting the expression of the LSD1 + 8a isoform [45]. SRRM4 can also activate a pluripotency gene network through the SRRM4-SOX2 signaling pathway, contributing to NEPC onset [76].

MicroRNAs (miRNAs) have recently emerged as significant regulators of NE plasticity. NEPC tumors are characterized by alterations in several miRNAs, including the downregulation of the miR-106a~363 cluster and miR-421. The miR-106a~363 cluster regulates multiple NEPC drivers, including MYCN, E2F1, STAT3 (Signal Transducer and Activator of Transcription 3) and AURKA [77]. Repression of miR-421 is induced by MYCN and ultimately activates ATM serine/threonine kinase expression [78], thus contributing to NE differentiation. On the other hand, upregulation of miR-194 and miR-147b is commonly observed in NEPC. miR-194 targets FOXA1 [79], while miR-147b targets the Ribosomal Protein S15a (RPS15A), which is downregulated in NEPC cells and inversely correlates with NE markers [80]. Additional miRNAs involved in NEPC are the miR-708, miR-106b~25 cluster, miR-32 and miR-204. Briefly, EZH2-mediated downregulation of miR-708 is associated with NE differentiation [81]; the overexpression of the miR-106b~25 cluster in response to hypoxia leads to the suppression of REST in NEPC cell lines [82]; mast cell infiltration following enzalutamide treatment upregulates the expression of miR-32 and promotes NE differentiation [83]; by targeting XRN1, miR-204 functions as an oncomiR in NEPC, reducing AR expression and enabling certain clones to acquire a more NE phenotype [84].

Regarding long non-coding RNAs (lncRNAs) and their role in NEPC, the conserved lncRNA LINC00261 binds to and sequesters miR-8485 in the cytoplasm, preventing it from targeting CBX2 (Chromobox 2) mRNA, a component of the PRC1 (Polycomb Repressive Complex 1) complex that is often upregulated in NEPC and required for NE differentiation [38,85]. In the nucleus, LINC00261 positively regulates the FOXA2 gene to drive NEPC proliferation and metastasis [86]. Recently, the driver of NEPC ONECUT2 has been shown to possess an extremely long 3′ untranslated region (3′ UTR) of 14,757 nucleotides that can operate, independently of the ONECUT2 protein, as a competitive endogenous RNA (ceRNA) to activate a neural lineage plasticity gene expression program that substantially overlaps with that of the ONECUT2 protein [87].

### 2.5. Altered Pathways and Biological Processes

Several signal transduction pathways have been identified as involved in driving the histologic transformation from prostate adenocarcinoma to NEPC. Notch pathway inhibition is a hallmark of NE tumors, which exhibit high expression of DLL3 (Delta-Like Protein 3), an inhibitory ligand of the Notch pathway, and high levels of ASCL1 (Achaete-Scute Family BHLH Transcription Factor 1), a transcriptional regulator of DLL3 [88,89]. Activation of the Wnt pathway contributes to the development of NEPC and aggressive tumor growth [64,90]. Unno et al. have shown that coactivation of ALK (Anaplastic Lymphoma Kinase) and MYCN is enough to transform mouse prostate basal stem cells into aggressive PC with NE differentiation by stimulating the Wnt/β-catenin pathway [91]. Another frequently activated signaling mechanism in NEPC is the PI3K/AKT pathway, which is commonly activated after PTEN loss [33,39,81,92].

Aberrantly expressed RET kinase acts as a driver of tumor growth in multiple models of NEPC [93]. The PKC (Protein Kinase C) family and their signaling pathways also promote NEPC. NRP1 (Neuropilin-1) is upregulated in response to ADT and activates the PKCδ isoform, promoting NE differentiation and drug resistance in PC [94]. The reduced expression of PKCλ/ι results in the upregulation of serine biosynthesis through the mTORC1 (Mammalian Target Of Rapamycin Complex 1)/ATF4 (Activating Transcription Factor 4) pathway. This metabolic reprogramming supports cell proliferation and increases intracellular SAM (S-adenosyl methionine) levels, facilitating epigenetic changes that impart NE characteristics to mCRPC [95]. Another metabolic feature of NE cells is their heightened glycolytic activity. These tumors exhibit high expression of the histone lysine demethylase KDM8, reprogramming metabolic genes towards aerobic glycolysis (Warburg effect) [96]. The cell surface protein CD44 and the plasma membrane lactic acid transporter MCT4 are also linked to glucose metabolism and have been shown to be essential for NEPC cell survival [97,98].

In NEPC, MYCN regulates the transcription of DDR (DNA damage response) genes, including the chromatin-associated enzyme PARP1 (Poly (ADP-Ribose) Polymerase 1) [99]. The stem cell marker TROP2 (Tumor-associated Calcium Signal Transducer 2) has been identified as a driver of the NE phenotype, with PARP1 acting as a key mediator of this effect [100].

Inhibition of the AR axis in PC cells leads to the induction of EMT, enrichment of CSC populations and NE differentiation [101]. Consequently, aberrant expression of some EMT-associated proteins is found in NEPC, including the transcription factors SNAI1 and SNAI2 (Snail Family Transcriptional Repressors 1 and 2), ZBTB46 (Zinc Finger and BTB Domain Containing 46) and some SRC family kinases, such as FYN [102,103,104,105].

### 2.6. Tumor Microenvironment

In addition to the intrinsic characteristics of tumors, components of the tumor microenvironment, such as immune cells, tumor-associated macrophages (TAMs), myeloid-derived suppressor cells (MDSCs) and cancer-associated fibroblasts (CAFs), can establish communications with PC cells and are critical in shaping PC progression [106]. PC cells secrete BMP6 (Bone Morphogenic Protein-6), which triggers the release of IL-6 (Interleukin 6) by TAMs [107], thus promoting NEPC through various pathways, including activation of the STAT3, TGF-β (Transforming Growth Factor Beta)/SMAD2 (SMAD Family Member 2) and MAPK (Mitogen-Activated Protein Kinase) pathways [108,109,110] and suppression of REST [111].

Recently, it has been observed that enzalutamide induces the expression of High Mobility Group Protein B1 (HMGB1), which facilitates the recruitment of TAMs and promotes NE differentiation via β-catenin stabilization. TAMs themselves secrete IL-6 and directly contribute to the promotion of HMGB1 expression, completing a feedback loop that reinforces the induction of NE characteristics [112]. Other TME factors such as hypoxia, neurotensin and tumor-derived exosomes containing Caveolin 1 (Cav-1) have been reported to be potent drivers of NE differentiation [65,66,113,114].

## 3. Potential Therapeutic Strategies Targeting NE Differentiation

In the era of evolving androgen-directed therapies, treating NEPC remains challenging because of broad therapy resistance. By effectively targeting the NE phenotype, it may be possible to suppress PC cell proliferation and inhibit NE differentiation, potentially delaying or reversing the development of androgen-independent PC. Several key factors are currently being targeted by a variety of small molecules and antibodies in pre-clinical and clinical studies (Table 1). Although there are currently no clinically approved precision drugs, progress is being made in several lines of investigation, and one or more of these approaches may help to overcome NEPC resistance and improve clinical management for PC patients.

### 3.1. Targeting Genomic Alterations

NEPC cell lines show enhanced sensitivity to AURKA inhibitors [32]. The ATP-competitive pan-Aurora kinase inhibitor Danusertib (PHA-739358) has demonstrated significant effects both in vitro and in vivo, inhibiting NEPC cell line proliferation and reducing tumor volume and NE activity in xenograft models [32,115]. However, a randomized phase II study showed minimal efficacy of Danusertib monotherapy in non-selected patients with mCRPC after docetaxel failure [116], possibly as ATP competitive inhibitors can leave the AURKA–MYCN complex unflawed [101,117]. On the contrary, the AURKA inhibitors Alisertib (MLN8237) and CD532 can effectively disrupt this complex, resulting in MYCN destabilization and cytotoxic activity in vitro [33,39,118]. Alisertib has been evaluated in a phase II clinical trial for NEPC treatment. While the trial did not achieve its primary endpoint of progression-free survival (PFS) in a biomarker-unselected population, two patients showed an exceptional response with complete eradication of liver metastases [119]. Ongoing pre-clinical studies are investigating the efficacy of other small molecules targeting aurora kinases, such as VX680, that have demonstrated potent anti-tumor activity in PC cell lines [120]. Additionally, Ton et al. have developed a molecule called 7082 that effectively targets both MYCN and AURKA, suppressing the proliferation of PC and NEPC cell lines [121]. The small molecule VPC-70619 specifically targets MYCN and has shown strong anti-proliferative activity against cell lines expressing MYCN, including NEPC cell lines. Pharmacokinetic studies have revealed that VPC-70619 exhibits high bioavailability through intraperitoneal and oral administration, making it a potentially valuable compound for the treatment of lethal NEPC [122].

Pharmacological targeting of factors upstream of MYCN and AURKA has been proposed as complementary therapeutic strategies for NEPC. NK1R (Tachykinin Receptor 1) activates the AURKA/MYCN signaling pathway through PKCα and its knockdown results in the reduction of tumor burden and suppression of NE features in vivo. Aprepitant, an FDA-approved selective NK1R antagonist, exerts anti-proliferative effects in NE-like and NEPC cells, and the PKC inhibitor GF109203X induces cell cycle G2/M arrest [118].

### 3.2. Targeting Epigenetic Factors

MYCN redirects EZH2 activity, and NEPC cells are sensitive to EZH2 inhibition [39]. A variety of EZH2 inhibitors are currently being tested in a wide range of cancers [123]. EZH2 inhibitors tested in pre-clinical studies include GSK343, GSK503, GSE126, DZNEP and EPZ6438 (Tazemetostat) [29,31,39,124]. Treatment with EZH2 inhibitors reduces MYCN–EZH2 interaction, results in downregulation of NE genes and can curb cell viability of NEPC cells [29,39,124]. Moreover, some of these inhibitors have demonstrated the ability to enhance AR expression and increase sensitivity to ADT in vitro and in vivo [31]. Based on these promising results, several clinical trials are currently investigating EZH2 inhibitor therapy alone or in combination with potent AR inhibitors for mCRPC (NCT03480646 and NCT04179864). Additional pre-clinical results demonstrate that GSK126 exhibits increased toxicity in NEPC cells when combined with the chemotherapy agent docetaxel [81], suggesting a potential novel treatment regimen to be evaluated.

In NEPC arising after ARSI therapy, EZH2 activity has been shown to be enhanced by the induction of the PKA/CREB (cAMP-response element binding protein) pathway. Of note, treatment with the β-adrenergic antagonist and PKA/CREB inhibitor Propranolol has been found to significantly reduce tumor growth, NE differentiation and angiogenesis in vivo [124].

PRC2 trimethylates H3K27, a histone mark recognized by the CBX subunit of the canonical PRC1 complex. Among the different CBX paralogs, CBX2 has been identified as a key player in NEPC progression [38,85]. A CBX2-specific chromodomain inhibitor named SW2_152F has been developed to target this pathway with promising results as it effectively blocks NE fate and promotes PC cell death [85].

The link between EZH2 and DNMTs has sparked interest in DNMTs as potential drug targets in NEPC. The hypomethylation agent decitabine reverts basal and NE markers and inhibits NEPC tumor growth in mice [95]. Its analog, azacytidine, has also been shown to partially re-sensitize ARSI-resistant NE-like PC cell lines [125]. Decitabine and azacytidine are already FDA-approved for the treatment of myelodysplastic syndromes and could be repurposed for NEPC treatment, although the latter showed weak anti-tumor activity in a phase II clinical trial in patients with mCRPC [126]. Currently, there are two trials in progress evaluating the combination of decitabine and guadecitabine (SGI-110) with enzalutamide and the immunotherapeutic drug pembrolizumab, respectively, in mCRPC patients (NCT05037500, NCT02998567).

Other drugs directed against epigenetic modulators have been investigated for targeting plasticity and the NEPC phenotype. NSD2 depletion using short hairpin RNAs (shRNAs) inhibits PC tumorigenicity both in vitro and in vivo [41]. Similar effects were obtained after treatment of DU145 xenografts with the small molecule inhibitor of NSD2 (MCTP-39) [41]. The epigenetic regulator LSD1, an emerging target for small cell lung cancer (SCLC) [127], has also been explored in NEPC. The allosteric inhibitors of LSD1, SP-2509 and SP-2577 are molecules that potentially could be used for NEPC treatment, as they effectively suppress NE cell growth and show good tolerability in in vivo models [44,128]. The reversible LSD1 inhibitor CC-90011 has been evaluated in a phase I clinical trial of advanced malignancies, including NEPC, and has shown an acceptable tolerability profile and promising overall clinical activity [129]. Currently, a clinical trial conducted exclusively in patients with mCRPC is trying to assess whether CC-90011 can induce AR expression and, consequently, re-sensitize tumors to anti-hormonal therapy (NCT04628988). 

Depletion of the chromatin modulator DEK with siRNAs (small interfering RNAs) suppresses cell growth, migration and invasion of PC3 cells, an AR-negative adenocarcinoma cell line sometimes used as an NEPC model [49]. DEK-targeted aptamers (DTAs) have been studied in the context of inflammatory arthritis [130] and could potentially be explored as a novel therapeutic approach for patients with NEPC.

Pre-clinical studies have revealed that BET inhibitors such as JQ1, ZEN-3694 and OTX-15 block the NE program and suppress NEPC growth by inhibiting the BRD4-E2F1 program [52] and MYCN-driven NE differentiation [131]. In a phase Ib/IIa clinical trial, ZEN-3694 in combination with enzalutamide has demonstrated acceptable tolerability and potential efficacy in patients with mCRPC, providing clinical evidence that BET inhibition may be able to abrogate resistance mechanisms and re-sensitize patients to AR-signaling inhibitors [132]. Other phase II clinical trials evaluating ZEN-3694 are currently active for mCRPC (NCT04471974, NCT04986423).

### 3.3. Targeting Transcription Factors

Although TP53 and RB1 mutations are almost universal in NEPC, they are not readily targetable. As an alternative strategy, there is growing interest in targeting common downstream effectors of both tumor suppressor genes. One such effector is PEG10, which has gained significant attention in recent studies. Targeting PEG10 using siRNAs or shRNAs effectively reduces the proliferation rate and expression of NE markers both in vitro and in vivo [60]. PEG10 possesses a unique ribosomal frameshift sequence and a protease domain similar to the HIV (human immunodeficiency virus), which makes it a suitable candidate for drug targeting. Another targetable transcription factor is ONECUT2, which has been shown to be a survival factor in mCRPC. Inhibition of ONECUT2 can be achieved using a small molecule named CSRM617 that reduces tumor size/weight and metastasis in xenograft models [67]. Additionally, the synergistic interaction between ONECUT2 and hypoxia has led to the investigation of an alternative therapeutic strategy involving the use of the hypoxia-activated prodrug TH-302, which reduces NEPC tumor growth in both xenograft and PDX (patient-derived xenografts) models [66].

Recent efforts have focused on the identification of inhibitors against the driver of lineage plasticity, BRN2. Deletion or stable knockdown of BRN2 prevents NE differentiation, thus reducing invasiveness and tumor proliferation in both enzalutamide- and castration-resistant PC [68]. While the first-in-field BRN2 inhibitor is developed, one approach to inhibit BRN2 signaling is by targeting its upstream regulator MUC1-C. Silencing MUC1-C leads to the downregulation of BRN2, decreasing the self-renewal capacity and tumorigenicity of PC cells [69]. Cell-penetrating peptides (CPPs), such as GO-203, block MUC1-C homodimerization and nuclear localization. GO-203 has already undergone evaluation in early-phase clinical trials for solid tumors (NCT01279603). However, its short half-life presents a challenge in the clinical setting, and new strategies, such as polymeric nanoparticle encapsulation (GO-203/NPs), are being explored [133]. In addition to CPPs, other approaches targeting the extracellular domain of MUC1-C have been investigated. Antibody-based approaches, including antibody–drug conjugates (ADCs) [134] and chimeric antigen receptor (CAR) T cells, have demonstrated potential in drugging the extracellular domain of MUC1-C. CAR-T cells targeting MUC1-C-expressing cancers are already undergoing phase I evaluation (NCT05239143), although no PC patients are included in this cohort.

Other strategies have been directed towards targeting FOX transcription factors. Paranjape et al. identified a critical nexus between p38MAPK signaling and FOXC2 for NEPC development [62]. This study has demonstrated that targeting FOXC2 using the p38 MAPK inhibitor, SB203580, can restore the epithelial phenotype and increase sensitivity to AR inhibition. Consequently, its combination with enzalutamide results in a substantial reduction of tumor growth in vivo [62]. FOXA2 knockdown induces the reversal of adeno-to-NE lineage transition [63]. As FOXA2 has been an elusive drug target, alternative strategies are being considered, such as inhibition of the SIAH2/HIF/FOXA2 axis. The FDA-approved drug menadione (Vitamin K3) is an inhibitor of SIAH2 that, in combination with ADT, delays the occurrence of mCRPC [135]. Another novel SIAH2 inhibitor, RLS-24, has demonstrated the ability to reduce PC cell viability [136]. These inhibitors are particularly relevant in the context of NEPC, as SIAH2 depletion with shRNAs results in marked suppression of NEPC tumors [65].

### 3.4. Targeting Pathways and Biological Processes

FOXA2 promotes NEPC by direct activation of KIT expression. Targeting the KIT pathway with tyrosine kinase inhibitors (TKIs), such as imatinib, sorafenib, sunitinib and cabozantinib, suppresses mouse and human NEPC tumor growth [63,137]. However, it is important to note that the TKI dovitinib has demonstrated unexpected effects, inducing NE differentiation instead of repressing it [138]. Various TKIs have been evaluated in clinical trials, yielding different efficacies. Notably, cabozantinib showed promising results in phase II trials [139,140] but did not significantly improve overall survival in phase III trials [141]. Cabozantinib is currently being assessed as monotherapy in a biomarker-selected subgroup of mCRPC (NCT04631744) as well as in combination with the checkpoint inhibitor nivolumab (NCT05502315) and atezolizumab (NCT04446117), among other clinical trials.

As a TKI, cabozantinib has the ability to block the RET kinase, an essential factor in NEPC development, as evidenced by the strong growth suppression of NEPC cell lines upon RET knockdown. Importantly, the molecules AD80, LOXO-292 and BLU-667 exhibit a higher degree of selectivity in inhibiting the RET pathway compared to cabozantinib and effectively induce cell death in NEPC 3D cultures and xenograft models, opening new possibilities for targeted therapies in NEPC treatment [93].

As above mentioned, SRC family kinases are also potential therapeutic targets in NEPC. Dasatinib (BMS-354825) is a Src/ABL TKI that has shown pre-clinical activity in PC cells [142]. Dasatinib demonstrated biological effects only in chemotherapy-naïve mCRPC patients [143,144], and the subsequent phase III study that combined dasatinib with docetaxel in mCRPC patients did not result in an improvement in overall survival compared to chemotherapy alone [145]. c-SRC also activates the MEK/ERK cascade to drive NE transdifferentiation from prostate adenocarcinoma [146]. The MEK1/2 inhibitor trametinib (TMT212) and the ERK inhibitor SCH772984 have shown anti-proliferative effects in human NE cell lines [147]. Trametinib is currently being evaluated in a phase II trial for patients with mCRPC (NCT02881242). In addition, SPHK1 (Sphingosine Kinase 1) plays an autocrine role in promoting NEPC transdifferentiation by activating ERK, eventually leading to REST proteasomal degradation. FDA-approved SPHK1-specific inhibitors, such as FTY720 or SKI-II, have demonstrated the ability to inhibit NEPC tumor growth and block REST protein degradation, resulting in reduced expression of NE markers in PDX models [148]. Recently, a theranostic small-molecule prodrug conjugate has been shown to be effective for precise delivery and intracellular release of FTY720 in NEPC cells [149].

PI3K/AKT inhibitors have also been explored to treat NE tumors. Pan-PI3K inhibitors, such as buparlisib (BKM-120) and dactolisib (BEZ235), which also inhibit mTOR, can effectively reduce cell viability in PC cells, particularly in those overexpressing MYCN. The pan-AKT inhibitors ipatasertib and MK2206 and the mTOR inhibitor RAD001 have also shown favorable pre-clinical results in mCRPC [39]. Several of these compounds blocking the PI3K/AKT pathway have been assessed in clinical trials. While some PI3K inhibitors like buparlisib, dactolisib or PX-866 did not show significant activity in patients with mCRPC [150,151,152], AKT inhibitors appear to be more promising; MK2206 demonstrated partial responses in two NEPC patients during a phase I trial [153], and ipatasertib combined with abiraterone has shown trends toward improved radiographical progression-free survival (rPFS) in mCRPC patients, particularly in cases with PTEN loss [154,155]. Another trial combining ipatasertib with chemotherapy and immunotherapy is ongoing for mCRPC (NCT03072238). Of note, two studies have provided insights into the effects of PI3K/AKT-targeted therapies, demonstrating that the PI3K inhibitor LY294002 can induce differentiation towards NEPC [156,157]. This finding highlights a potential risk associated with the AR and PI3K/AKT co-targeting strategy.

The Wnt pathway can be blocked with the small molecule inhibitor LGK974, which is undergoing a phase I clinical trial in a wide range of solid tumors (NCT01351103). Bland et al. demonstrated that LGK974 treatment effectively inhibits NEPC tumor growth and reduces the expression of the NE marker CD56 both in vitro and in vivo [90]. Other molecules tested in NEPC cells include the Wnt inhibitor ICG-001 and the β-catenin inhibitor XAV-939 [91]. Notably, ICG-001 showed an additive effect in combination with the ALK inhibitor alectinib, leading to the suppression of NEPC proliferation in vitro and the inhibition of tumor growth and metastasis in vivo [91].

Given the high similarities between NEPC and SCLC, DLL3-targeted therapies employed in SCLC are also being studied in the context of NEPC [158]. DLL3+ NEPC xenografts have been shown to be sensitive to rovalpituzumab tesirine (SC16LD6.5), a DLL3-targeted ADC [88]. In a phase I basket trial, a patient with DLL3-expressing NEPC experienced a significant reduction of nodal metastases upon treatment with this drug (NCT02709889). Ongoing studies are investigating other antibodies targeting DLL3 in NEPC, including the bispecific antibodies tarlatamab (NCT04702737) and PT217 (NCT05652686).

Among the EMT/NEPC-associated factors, ZBTB46 lacks specific inhibitors directly targeting its activity. Thus, efforts are being made to target downstream effectors of ZBTB46, such as LIF (Leukemia Inhibitory Factor), PTGS1 (Prostaglandin G/H Synthase 1) and NGF (Nerve Growth Factor)/CHRM4 (Cholinergic Receptor Muscarinic 4) [159,160,161]. The LIF inhibitor EC330 [159], the PTGS1 inhibitor NS-398 [160], the NGF inhibitor RO08-2750 [161] and the CHRM4 inhibitor ceritinib [162] have shown efficacy in suppressing tumor growth and NE differentiation in PC. Knockdown of SNAI1 can block NE differentiation [102], and its inhibition can be achieved by the novel proteasome inhibitor NPI-0052 (salinosporamide A) [163,164]. MLN4924 (pevonedistat), a small molecule currently undergoing phase II clinical trials for cancer, inhibits SNAI2 [165] and, when combined with ADT or ARSIs, significantly enhances growth suppression of PC [166]. Importantly, it has been shown that MLN4924 suppresses SOX2 expression [167], a particularly relevant target in the context of NEPC.

Another novel therapeutic strategy for NEPC consists of targeting the MYCN–PARP–DDR pathway [115]. The PARP1 inhibitors talazoparib and olaparib can reverse the NE phenotype induced by TROP2 in PC cells and decrease tumor growth in TROP2-expressing NEPC xenografts [100]. The possibility of co-targeting AURKA and PARP has also been studied. Inhibition of AURKA with PHA739358 and olaparib successfully suppressed growth in pre-clinical studies [115]. Additionally, PARP1 inhibitors have been tested together with CDK4/6 inhibitors, which suppress E2F1 signaling frequently found activated in NEPC. The combination of olaparib and the FDA-approved CDK4/6 inhibitors palbociblib or abemaciclib results in the suppression of NE markers and tumor growth [168]. Similar results have been observed when combining olaparib and dinaciclib, a CDK2/5 inhibitor [169].

Regarding the NRP1/PKC pathway, inhibition of NRP1 protein expression or suppression of PKC activation leads to the inhibition of NE differentiation and prevents tumor progression towards castration resistance. Enzastaurin, a potent pan-PKC inhibitor, can reduce the expression of NE markers in LNCaP-NE cells and enhance the cytotoxic effects of docetaxel in NEPC cells in in vitro and in vivo models [94].

As above mentioned, molecular events driven by metabolic reprogramming are one of the key hallmarks of NEPC. Consequently, emerging therapeutic strategies are being tailored to target genes intricately associated with metabolic pathways such as GGPS (Geranylgeranyl Pyrophosphate Synthase) and HK2 (Hexokinase 2). Essential genes in the isoprenoid pathway are highly expressed in NEPC, and geranylgeranylation of proteins contributes to the development of the NE phenotype. The novel compound DGBP (digeranyl bisphosphonate), which acts as a selective inhibitor of GGPS, has shown promise in inhibiting the progression of PC to its NE form [170]. Lastly, TRIM36 (Trigred Motif 36) is lowly expressed in NEPC due to its inhibitory role in glycolysis via the ubiquitination of HK2. Treating PC cells with the glycolysis inhibitor 2DG (2-deoxy-d-glucose) or the HK2 inhibitor 3BP (3-bromopyruvate) inhibits glycolysis, promotes ferroptosis and suppresses NE differentiation [171].

### 3.5. Targeting Post-Transcriptional Regulators

Several approaches have been investigated to inhibit splicing factors and post-transcriptional regulators involved in NEPC. Blocking SRRM4 with a specific antisense oligonucleotide (ASO) results in a significant decrease in SCLC and PC cell viability [172]. The RNA-binding protein LIN28B can also be targeted with a series of small molecule inhibitors (Ln7, Ln15 and Ln115) that have shown the ability to block CSC characteristics and suppress SOX2 expression [173]. Inhibition of miR-147b, miR-194 and miR-32 leads to the reversal of NE features and suppresses the growth of PC cell lines [79,80,83]. As mentioned before, MYCN regulates the miR-421/ATM pathway to promote the development of therapeutic resistance in NEPC. Inhibition of ATM with the small molecule Ku60019 in combination with Enzalutamide re-sensitizes MYCN-expressing cells to AR inhibition and prevents metastasis, suggesting a promising novel treatment regimen for NEPC to be explored [78].

### 3.6. Targeting the TME

Secretion of MIF (macrophage migration inhibitory factor) during NEPC has been shown to facilitate cancer progression and recurrence. Thus, MIF represents an attractive target for aggressive PC treatment that can be inhibited with the antagonist ISO-1, which disrupts the MIF–CD74 interaction [174,175]. Inhibitors of the IL-6/JAK/STAT3 signaling cascade, such as siltuximab (anti-IL6), P6 (pan-JAK inhibitor), galiellalactone (anti-STAT3) and LLL12 (which blocks STAT3 phosphorylation), have also been studied in PC [176,177]. Despite showing promising biological activity in in vitro studies, the clinical efficacy of the monoclonal antibody siltuximab was found to be limited in a phase II clinical trial involving patients with mCRPC [178]. IL-6 also activates the TGF-β/SMAD2 axis to confer NE properties to PC cells. Targeting TGF-β with galunisertib (LY2157299) and LY364947 failed to attenuate NE differentiation in PC cells under ADT, although its combination with the p38 MAPK inhibitor SB203580 effectively inhibits the NE phenotype in vitro [109].

**Table 1:** Novel targeted therapies under development for mCRPC/NEPC.

Target	Drug	Study Phase	Ref.
AURKA	Danusertib (PHA-739358)	Pre-clinical (in vitro and in vivo). Clinical trial phase II completed	[32,115,116]
	Alisertib (MLN8237)	Pre-clinical (in vitro). Clinical trial phase II completed	[33,39,118,119]
	CD532	Pre-clinical (in vitro and in vivo)	[33]
	VX680	Pre-clinical (in vitro)	[120]
	7082	Pre-clinical (in vitro)	[121]
MYCN	7082	Pre-clinical (in vitro)	[121]
	VPC-70619	Pre-clinical (in vitro)	[122]
NK1R	Aprepitant	Pre-clinical (in vitro)	[118]
EZH2	GSK343	Pre-clinical (in vitro)	[29,39]
	GSK503	Pre-clinical (in vitro and in vivo)	[31,39]
	GSK126	Pre-clinical (in vitro)	[31,39,81,124]
	DZNEP	Pre-clinical (in vitro)	[124]
	EPZ6438 (Tazemetostat)	Pre-clinical (in vitro). Clinical trial phase Ib/II ongoing	[31], NCT04179864
	CPI-1205	Clinical trial phase Ib/II ongoing	NCT03480646
CBX2	SW2_152F	Pre-clinical (in vitro)	[85]
DNMT	Decitabine	Pre-clinical (in vitro and in vivo). Clinical trial phase I ongoing	[95], NCT05037500
	Azacytidine	Pre-clinical (in vitro). Clinical trial phase II completed	[125,126]
	Guadecitabine (SGI-110)	Clinical trial phase I ongoing	NCT02998567
PKA/CREB	Propranolol	Pre-clinical (in vitro and in vivo)	[124]
NSD2	MCTP-39	Pre-clinical (in vitro and in vivo)	[41]
LSD1	SP-2509	Pre-clinical (in vitro)	[44,128]
	SP-2577 (Seclidemstat)	Pre-clinical (in vitro and in vivo)	[44]
	CC-90011	Clinical trial phase I ongoing	[129], NCT04628988
DEK	DEK-targeted aptamers	Not tested in PC models	[49,130]
BET	JQ1	Pre-clinical (in vitro and in vivo)	[52,131]
	OTX-15	Pre-clinical (in vitro)	[131]
	ZEN-3694	Pre-clinical (in vitro). Clinical trial phase Ib/IIa completed and phase II ongoing	[52,132], NCT04471974, NCT04986423
ONECUT2	CSRM617	Pre-clinical (in vitro and in vivo)	[67]
Hypoxia	TH-302	Pre-clinical (in vitro and in vivo)	[66]
MUC1-C	GO-203, ADCs, CAR-T	Not tested in PC models	[69,133,134]
p38 MAPK	SB203580	Pre-clinical (in vitro and in vivo)	[62,109]
SIAH2	Menadione	Pre-clinical (in vitro and in vivo)	[135]
	RLS-24	Pre-clinical (in vitro)	[136]
KIT	Imatinib, Sorafenib, Sunitinib	Pre-clinical (in vitro)	[63]
	Dovitinib	Pre-clinical (in vitro and in vivo)	[138]
	Cabozantinib	Pre-clinical (in vitro and in vivo). Clinical trials phase II and III completed and other phase II and III ongoing	[63,137,139,140,141], NCT04631744, NCT04446117, NCT05502315
RET	Cabozantinib	Pre-clinical (in vitro and in vivo). Clinical trials phase II and III completed and other phase II and III ongoing	[63,137,139,140,141], NCT04631744, NCT04446117, NCT05502315
	AD80	Pre-clinical (in vitro and in vivo)	[93]
	LOXO-292, BLU-667	Pre-clinical (in vitro)	[93]
SRC signaling	Dasatinib (BMS-354825)	Pre-clinical (in vitro and in vivo). Clinical trials phase II and III completed	[142,143,144,145]
MEK/ERK	Trametinib (TMT212)	Clinical trial phase II ongoing	NCT02881242
	SCH772984	Not tested in PC models	[147]
SPHK1	FTY720, SKI-II	Pre-clinical (in vitro and in vivo)	[148]
PI3K/AKT/mTOR	Buparlisib (BKM-120)	Pre-clinical in vitro. Clinical trial phase II completed	[39,150]
	Dactolisib (BEZ235)	Pre-clinical in vitro. Clinical trial phase I/II completed	[39,151]
	PX-866	Clinical trial phase II completed	[152]
	LY294002	Pre-clinical (in vitro)	[156,157]
	Ipatasertib	Pre-clinical (in vitro). Clinical trials phase II and III completed and other phase III ongoing	[39,154,155], NCT03072238
	MK2206	Pre-clinical (in vitro). Clinical trial phase I completed	[39,153]
	RAD001	Pre-clinical (in vitro)	[39]
Wnt signaling	LGK974	Pre-clinical (in vitro and in vivo)	[90,91]
	ICG-001	Pre-clinical (in vitro and in vivo)	[91]
	XAV-939	Pre-clinical (in vitro)	[91]
ALK	Alectinib	Pre-clinical (in vitro and in vivo)	[91]
DLL3	Rocalpituzumab tesirine (SC16LD6.5)	Pre-clinical (in vitro and in vivo). Clinical trial phase I completed	[88], NCT02709889
	Tarlatamab	Clinical trial phase I ongoing	NCT04702737
	PT217	Clinical trial phase I ongoing	NCT05652686
LIF	EC330	Pre-clinical (in vitro and in vivo)	[159]
PTGS1	NS-398	Pre-clinical (in vitro and in vivo)	[160]
NGF	RO08-2750	Pre-clinical (in vitro and in vivo)	[161]
CHRM4	Ceritinib	Pre-clinical (in vitro and in vivo)	[162]
SNAI1	NPI-0052 (Salinosporamide A)	Pre-clinical (in vitro)	[163,164]
SNAI2	MLN4924 (Pevonedistat)	Pre-clinical (in vitro and in vivo)	[166]
PARP1	Talazoparib	Pre-clinical (in vitro and in vivo)	[100]
	Olaparib	Pre-clinical (in vitro and in vivo)	[100,115,168,169]
PKC	Enzastaurin	Pre-clinical (in vitro and in vivo)	[94]
	GF109203X	Pre-clinical (in vitro)	[118]
GGPS	DGBP	Pre-clinical (in vitro)	[170]
Glycolysis	2DG	Pre-clinical (in vitro)	[171]
HK2	3BP	Pre-clinical (in vitro)	[171]
SRRM4	ASO	Pre-clinical (in vitro)	[172]
LIN28B	Ln7, Ln15, Ln115	Pre-clinical (in vitro)	[173]
miR-147b	anti-miR-147b	Pre-clinical (in vitro)	[80]
miR-194	miR-194 LNA inhibitor	Pre-clinical (in vitro)	[79]
miR-32	miRNA32 inhibitor	Pre-clinical (in vitro)	[83]
ATM	Ku60019	Pre-clinical (in vitro)	[78]
MIF	ISO-1	Pre-clinical (in vitro and in vivo)	[174,175]
IL6/STAT3	Siltuximab (CNTO 328)	Pre-clinical (in vitro). Clinical trial phase II completed	[176,178]
	LLL12	Pre-clinical (in vitro and in vivo)	[176]
	Galiellalactone	Pre-clinical (in vitro and in vivo)	[177]
	P6	Pre-clinical (in vitro)	[176]
TGF-β	Galunisertib (LY2157299)	Pre-clinical (in vitro)	[109]
	LY364947	Pre-clinical (in vitro)	[109]

ADCs: antibody–drug conjugate; CAR-T: chimeric antigen receptor T cell; ASO: antisense oligonucleotides; LNA: locked nucleic acid.

## 4. Conclusions and Future Directions

The rise of NEPC incidences in recent years is believed to be associated with the selective pressure exerted by potent drugs targeting the AR pathway. As a result, there is a growing need to deepen our understanding of the mechanisms contributing to the emergence of NEPC. The identification of key molecular pathways and factors involved in NEPC has provided new opportunities for targeted therapeutic interventions. Novel molecules are being proposed and tested as potential therapeutic strategies for this lethal disease, holding promise for improving outcomes and promoting precision medicine in the management of NEPC patients and highlighting the great necessity to develop biomarker-driven clinical approaches.

## Data Availability

Not applicable.
